# COVID-19 Booster Vaccination Status and Long COVID in the United States: A Nationally Representative Cross-Sectional Study

**DOI:** 10.3390/vaccines12060688

**Published:** 2024-06-20

**Authors:** Jamie L. Romeiser, Kelsey Schoeneck

**Affiliations:** Department of Public Health and Preventive Medicine, Upstate Medical University, 766 Irving Ave., Syracuse, NY 13210, USA; schoenek@upstate.edu

**Keywords:** COVID-19, vaccines, booster vaccination, long COVID, severe infection

## Abstract

Early studies have found that the initial COVID-19 vaccination series was protective against severe symptoms and long COVID. However, few studies have explored the association of booster doses on severe disease outcomes and long COVID. This cross-sectional analysis used data from the 2022 US National Health Interview Survey data to investigate how vaccination status correlates with COVID-19 infection severity and long COVID among previously infected individuals. Participants were categorized into three groups: those who had received at least one booster, those with only the initial complete vaccination series, and those with either an incomplete series or no vaccinations. Out of 9521 survey respondents who reported a past positive COVID-19 test, 51.2% experienced moderate/severe infections, and 17.6% experienced long COVID. Multivariable regression models revealed that receiving at least one booster shot was associated with lower odds of experiencing moderate/severe symptoms (aOR = 0.78, *p* < 0.001) compared to those unvaccinated or with an incomplete series. Additionally, having at least one booster reduced long COVID odds by 24% (aOR = 0.76, *p* = 0.003). Completing only the primary vaccine series did not significantly decrease the likelihood of severe illness or long COVID. These findings support the continued promotion of booster vaccinations to mitigate long COVID risks in vulnerable populations.

## 1. Introduction

The coronavirus disease (COVID-19) is caused by severe acute respiratory syndrome coronavirus 2 (SARS- CoV-2) [1] and has an estimated mortality rate of 2–6.6% [2,3] depending on global location and variant [3,4]. As of December 31, 2023, the number of confirmed COVID-19 cases has surpassed 770 million, with nearly 7 million confirmed deaths confirmed globally [5]. To date, approximately 13.4% of all cases (exceeding 103 million) and 16.4% of fatalities (surpassing 1.1 million) occurred in the United States alone [6]. In 2021, the United Health Foundation’s Health Rankings Annual Report found that COVID-19 was the third-leading cause of death in the U.S. [7]. While the age-adjusted COVID-19 mortality rates in the U.S. have declined by almost half in 2022 compared to 2021 [8], the virus remains a significant and persistent public health concern.

Part of the enduring public health challenge is understanding who may be at greater risk for developing both severe initial symptoms and a prolonged duration of symptoms associated with a COVID infection. A more severe initial infection is thought to lead to a higher likelihood of developing a post-COVID-19 condition (PCC), or long COVID [9,10]. The World Health Organization (WHO) defines a PCC as the occurrence of symptoms three months after the onset of the COVID-19 infection, persisting for a minimum of two months, and it cannot be explained by an alternative diagnosis [9,10]. Long COVID manifests in multiple organs with diverse symptoms, and many aspects of this condition remain poorly understood [11]. Common clinical symptoms include dyspnea, pain, fatigue, autonomic dysfunction, cognitive impairment, headache, deep vein thrombosis, gastrointestinal dysfunction, and persistent loss of smell or taste [9,12].

Prevalence estimates of long COVID tend to vary depending on both the definition and timing of the sampling frame. Recent estimates from the 2023 U.S. Census Bureau’s Household Pulse Surveys found that the trends are stabilizing to around 1 in 10 adults who have previously tested positive for COVID-19 [13]. Several studies have found that prevalence was lower during periods dominated by the Epsilon or Omicron variants and is highest amongst females and in those above 40 [11,13]. A recent meta-analysis identified other risk factors for post-COVID conditions including female sex, older age, obesity, smoking, various comorbidities such as anxiety, asthma, COPD, diabetes, immunosuppression, ischemic heart disease, and severity of initial symptoms [9].

Vaccinations have been shown to be protective against severe symptoms [2] and long COVID [9,12,14], but the impact may depend on timing [15] and the number of vaccinations. Three meta-analyses that used data from studies conducted throughout 2022 found that completion of an initial vaccination series (two doses) significantly reduced the risk of PCC by 29% [10], 36% [14], and 43% [9] compared to non-vaccinated individuals. A large U.S. national cohort study that monitored participants through mid-2022 [12], and a large cross-sectional study both found that a complete initial vaccination series prior to infection reduced the odds of long COVID by about 30% [11,12]. Nearly all of these studies found that a single vaccination dose offered less or no protection against long COVID.

Notwithstanding, vaccine effectiveness can wane over time even with a complete initial dose [1,10]. With the constant evolution of the coronavirus, booster doses have demonstrated the ability to restore protection against infection [16,17]. However, to date, there are few population-level studies that have specifically examined the associations between booster doses, severe disease sequelae, and long COVID. To address this question, we used data from the 2022 National Health Interview Study (NHIS) to investigate individuals who had received booster vaccinations, an initial complete vaccination series only, an incomplete initial vaccination, or were unvaccinated. Specifically, amongst individuals who have had a previous COVID-19 infection, we examined the associations between vaccination status and a severe COVID-19 infection, and vaccination status and occurrence of long COVID.

## 2. Materials and Methods

**Data Source:** In this cross-sectional study, we used data from the 2022 National Health Interview Survey (NHIS) [18]. The NHIS was initiated in 1957 and has been conducted by the National Center for Health Statistics (NCHS) since 1960 [19]. The primary goal of the NHIS is to monitor the health of the population through data collection and analysis of health-related topics by interviewing a nationally representative sample of U.S. households. NHIS includes annual content that appears on the survey every year as well as rotating core content, sponsored content, and emerging content that are included periodically [19]. Interviews are usually conducted in person, but follow-ups to complete interviews by telephone are common, with 55.7% of sample adult interviews in 2022 being at least partially conducted over the phone [19]. Similar to other nationally representative databases, NHIS oversamples certain subpopulations to increase the reliability and precision of health indicator estimates for these particular groups. Sample weights are then applied to the data to reflect the true U.S. population. More information about the NHIS sampling techniques and data collection can be found on the CDC website, https://www.cdc.gov/nchs/nhis/2022nhis.htm (accessed on 15 August 2023). All data are publicly available and de-identified and, therefore, did not require IRB review under the federal regulations for human subjects.

**Study Population:** The inclusion criteria for this study were defined as adult respondents who reported testing positive for COVID-19 (responded yes to the question, “Did you ever take a test that showed you had coronavirus or COVID-19?”) and provided responses for the vaccination and symptom questions used to define the primary outcomes and predictors.

**Primary Outcomes:** Two primary outcomes were examined. First, symptom severity was assessed using the following question: “How would you describe your coronavirus symptoms when they were at their worst? Would you say no symptoms, mild symptoms, moderate symptoms, or severe symptoms?” Symptoms were dichotomized into asymptomatic/mild and moderate/severe [20]. The second outcome examined the occurrence of long COVID, assessed by the following question: “Did you have any symptoms lasting 3 months or longer that you did not have prior to having coronavirus or COVID-19?”.

**Primary Predictor:** The primary predictor variable was vaccination status, which was defined as those who reported no/incomplete initial dosing, those with a complete initial dose/series only, and those who received both the complete initial series as well as at least 1 booster. Three questions were used to operationalize this primary predictor variable: “Have you had at least one dose of a COVID-19 vaccination?” and “How many COVID-19 vaccinations have you received?” were combined to develop a count of COVID-19 vaccinations from 0 to 4 or more. The question “For your first shot, which brand of COVID-19 vaccine did you receive?” was also used. Those who reported 0 vaccines or only 1 vaccine from a two-dose vaccination brand (“Pfizer”, “Moderna”, “Novavax”, or “One brand that required 2 doses”) were classified as having no/incomplete dosing. We chose to combine no/incomplete dosing groups into our reference category for two reasons. First, evidence from a systematic review suggests there may be limited protection offered against long COVID from just 1 vaccine dose [21]. Second, the number of individuals who reported only 1 dose from a 2-dose brand was very small and did not differ in outcomes from those with 0 doses. Those who received 2 doses from a 2-dose brand, or those reporting only 1 dose from a 1-dose brand (Janssen) were classified as having complete initial dosing. All individuals who reported a total number of vaccinations of 3 or more were classified as having at least 1 booster. Finally, those who received Janssen as an initial brand, but reported at least 2 vaccinations were classified as having at least 1 booter.

**Covariates:** Risk factors associated with developing severe symptoms or a long COVID were identified [9]. Covariates considered for analysis included respondents’ age (categorized into decades), sex (male, female), race (White, Black/African American, Asian, Hispanic, Other/Multiple), family income as a percentage of the federal poverty level (PIR, <1.25, 1.25 < 3.0, ≥3.0), education level (high school degree or less, some college, associate’s degree, bachelor’s degree, graduate degree), body mass index (BMI categorized as underweight, healthy weight, overweight, and obese), smoking history (ever smoked or not) and prior medical conditions including anxiety, depression, COPD, current asthma, diabetes, and immunocompromised status.

**Analysis:** All variables were described as frequencies and percentages. To account for the multi-stage sampling technique used by NHIS, survey weights were applied to all data analyses to obtain national estimates for the overall U.S. population. Weighted proportions are described throughout the narrative, but both weighted and unweighted proportions are reported. Differences in the distribution of demographic and comorbid conditions by vaccination status were assessed using chi-square tests. Weighted bivariate logistic regressions were performed to assess unadjusted associations between vaccine status, covariates, and both outcomes. Two weighted multivariable logistic regression models examined the adjusted association of vaccination status and symptom severity and long COVID. Multicollinearity among predictor variables was not detected, as all variance inflation factor (VIF) values were less than two. Because COVID-19 symptoms were included as a covariate for predicting long COVID, we also examined the potential overlapping distribution of long COVID amongst the symptom groups to ensure adequate separation between these two variables. We also examined the potential of effect modification between vaccination status and COVID symptom severity on the likelihood of developing long COVID, but no significant interaction was found.

Sensitivity analyses were performed by considering the number of COVID vaccination shots participants received as a continuous variable, and as a categorical variable without considering the brand of vaccination (0 shots, 1–2 shots, 3 or more shots). The results of these analyses were similar to the primary findings. An additional sensitivity analysis was performed that excluded asymptomatic individuals from the long-COVID analysis, and there were no discernable differences in the effects of the primary vaccine status variable. Additional sensitivity analyses are detailed in the discussion section. Missing data was less than 5% total and, therefore, no values were imputed. All analyses were performed using SAS 9.4 Software (Cary, NC) at the *p* = 0.05 level.

## 3. Results

A total of 27,651 NHI surveys were collected in 2022. Participants were excluded from our analysis if they never tested positive for COVID-19 (n = 17,875 [64.6%]) or did not have data for initial infection severity of symptoms (n = 8 [<0.01%]), long COVID (n = 73 [0.3%]), or vaccination data (n = 174 [0.6%]). A total of 9521 (34.4%) met the inclusion criteria, which equated to a weighted frequency representing 95.2 million U.S. citizens. Around 73% were white, 52.5% were female, and around 44.6% were under the age of 39 (Table 1).

The data showed that 23.9% of participants received either no COVID-19 vaccination or an incomplete initial dosing, 33.6% received complete initial dosing only, and 42.5% received at least one additional booster. A higher number of doses was associated with increasing age, PIR, and education (all *p* < 0.001). Within the boosted group, there were higher proportions of females and those with a diagnosis of diabetes or who were immunocompromised. There were higher proportions of smokers and obesity within the incomplete or no vaccine group (Table 1).

Symptom severity was also associated with vaccination status, with a higher proportion of the boosted group reporting asymptomatic or mild symptoms from their COVID-19 infection (*p* < 0.001). Further, the prevalence of long COVID was 17.6%, and significantly associated with booster status. Amongst the boosted group, 15.5% reported experiencing long COVID, whereas 20.1% of the no/incomplete vaccine group reported experiencing long COVID (*p* < 0.001).

Unadjusted and adjusted analyses predicting symptom severity are presented in Table 2 and in Table 3 for long COVID. Most effects were similar in the unadjusted and adjusted model results for symptom severity. After adjustment for age, sex, race, PIR, education level, smoking history, BMI, and prior medical conditions, those who received at least one booster vaccination were less likely to report moderate/severe symptoms from an initial COVID-19 infection compared to the no/incomplete vaccination group (adjusted OR = 0.78, *p* < 0.001). Those who received just the complete initial dose demonstrated a slightly protective yet insignificant effect (aOR = 0.95, *p* = 0.46). Similar results were seen when examining the outcome of long COVID. In the adjusted model, those who received at least one booster vaccination were less likely to report experiencing long COVID compared to those who were in the no/incomplete vaccination group (aOR = 0.76, *p* = 0.003). Of note, symptom severity was also highly associated with long COVID. Approximately 29% of those who experienced moderate/severe symptoms experienced long COVID, compared to 7% of those who were asymptomatic or had mild symptoms (aOR = 3.88, *p* < 0.001). An interaction term was examined for symptom severity and vaccination status but was not significant.

A sensitivity analysis demonstrated consistency when considering the number of COVID shots received as a continuous variable (0–4). After adjustment for all covariates, for every additional shot received, the odds of experiencing moderate/severe symptoms significantly decreased by about 7% (aOR = 0.93, 95% CI 0.89–0.97, *p* = 0.001), and the odds of long COVID significantly decreased by about 8% (aOR = 0.92, 95% CI 0.86–0.97, *p* = 0.003). A final sensitivity analysis examined the number of shots recategorized as no shots, 1–2 shots, and 3–4 shots and revealed similar findings to the initial analysis.

## 4. Discussion

Among a nationally representative group of U.S. adults who have previously tested positive for COVID-19, we found that COVID-19 vaccination status, specifically the reception of at least one booster dose, was associated with a significantly reduced likelihood of experiencing both severe initial COVID-19 symptoms and long COVID. Interestingly, completion of an initial primary COVID-19 vaccination series alone was not associated with reduced odds of severe disease or long COVID.

These results are in line with earlier studies that have found a protective effect of vaccinations against the risk of developing long COVID [9,10,11] and experiencing severe illness [1,2,22]. However, we no longer saw a protective association for an initial complete series alone. It is known that vaccine efficacy can wane over time [1,23]. Failure of partial immunization to protect individuals from long COVID has also been previously reported [9,10,11].

Consistent with our findings, other studies have found that booster vaccination doses increase protection against severe cases of COVID [24]. Severe infections have been linked to long COVID [9,10,20], which was also consistent with our findings. Having a moderate to severe infection had the largest effect size in our model for long COVID. Additional boosters appear to provide additional protection and are likely required in order to maintain a decreased individual risk for long COVID [25]. Few studies have examined the relationship between booster vaccinations and long COVID, but one case control study in Brazil found that individuals with 4 doses prior to any COVID infection were less likely to experience long COVID [26]. Another cohort study of healthcare workers in Italy found 3 doses to be protective against long COVID [27].

In this study of individuals with a prior COVID infection, the prevalence of long COVID was 17%. This is lower than a 2021 national estimate of almost 30% [7], but similar to the rate of 15% reported by another U.S. national cross-sectional study that was conducted from February 2021 to July 2022 [11]. Additional data have confirmed a general decline in the prevalence of post-COVID conditions amongst those with a prior infection from 19% in June 2022 to 11% in January 2023 [13]. This could be related to an increase in vaccination rates over time or some level of survivor bias. In general, the probability of long COVID was found to be less likely during the periods of Epsilon or Omicron variant predominance compared with the ancestral viral strain [11]. Care must be exercised when comparing different data that measure long COVID due to potential differences in definitions [28]. Long COVID as assessed within the NHIS survey likely meets the definition of long COVID utilized by the US Health and Human Services (signs/symptoms present for at least 4 weeks after the initial infection), but may not meet the definition used by the WHO (symptoms 3 months after the onset of COVID-19 infection, persisting for a minimum of 2 months).

There were additional findings from our models that are worth noting. Most of the findings in both of the adjusted models were similar. For example, those who were immunocompromised and those diagnosed with anxiety or asthma had a higher likelihood of having a more severe infection and experiencing long COVID. Gender disparities exist for both outcomes, with females having 44% higher odds of long COVID, and 27% higher odds of experiencing severe illness compared to males. A higher risk of long COVID among females has been observed in multiple preceding studies [7,9,11,12]. Disparities between racial and ethnic groups in the sample were also noted for both outcomes. Compared with non-Hispanic White adults, those with Hispanic ethnicity were more likely to report moderate/severe COVID-19 symptoms by 27%. An increase in symptom severity and mortality among Hispanic populations was found in previous research [7,29]. The increase in the likelihood of long COVID for Hispanic adults in this study was not determined to be statistically significant; however, a significant increase has been described in prior studies [7,11]. Non-Hispanic Black and Asian adults in our study had significantly reduced the odds of developing long COVID. Prior research has reported a similar significant decrease in long COVID among Asian populations [7,11]. Findings on the threat of long COVID to Black populations have varied, showing either decreased or similar odds of long COVID, when compared with White populations [7,11,30]. Obesity increased the odds of long COVID by 49% and severe illness by 20% compared to individuals with a healthy body weight. The association of obesity’s impact on increasing the likelihood of severe illness, hospitalization, and death was reported by Piernas et al. and explored in detail [22]. Increasing age was a significant predictor of long COVID, supporting previous research [7,11,26,27]. Age was not associated with symptom severity, contradicting prior reports [1,4]. Notably, one study found that age was no longer significantly associated with symptom severity, after adjusting for the patient’s health insurance status [29]. Conversely to age, increasing poverty income ratio and diabetes were both associated with symptom severity, but not with long COVID.

Targeted booster interventions could be helpful for those who are at higher risk for long COVID, particularly those with clinical conditions such as anxiety, obesity, COPD, asthma, and who are immunocompromised. However, pandemic fatigue is high, and booster uptake in the U.S. has stalled. One survey on booster receipt was distributed from February to March 2023 to an Arizona cohort who had received at least one prior vaccination in the past. The most common reason cited for not obtaining an updated booster vaccination was the occurrence of a prior SARS-CoV-2 infection [31]. A national survey conducted around that same time found that individuals who had a breakthrough infection were more likely to perceive vaccines as less effective [32].

Natural immunity acquired through a past infection can offer protection against reinfection as well as hospitalization [33]. As noted in one Swedish retrospective cohort study, however, hybrid immunity (vaccination after an infection) compared to natural immunity appears to provide an additional layer of protection against both reinfection and hospitalization [34]. More longitudinal research is needed to understand how boosters received after an infection may protect against the development of long COVID from a future infection. For those already experiencing ongoing long-COVID symptoms, obtaining additional vaccinations may not provide symptom relief [14,35], but not all studies agree [10,36]. A recent study found that receiving a vaccination while experiencing PCC decreased the number of symptoms as well as systemic inflammation [36].

Our study has several strengths. We used a large sample size of over 9500 survey respondents. Further, these data reflect the general U.S. population. We were able to account for numerous sociodemographic and comorbid risk factors for long COVID and further illustrate the need for targeted prevention within these subgroups. We were also able to use the brand of initial vaccination to help differentiate between a complete and incomplete initial series.

Our study has several limitations. First, this is a cross-sectional study. Our data cannot establish time-ordered events and, therefore, cannot establish causality. It’s likely that some participants were vaccinated after a COVID-19 infection. Individuals within this survey who were vaccinated after experiencing severe symptoms or long COVID would drive results towards the null. It is difficult to determine what effect the prior severity of infection has on COVID-vaccine intent, as the previous literature on the subject has been mixed [37,38]. Further, detailed information on vaccine timing and brand were not ascertained for every shot, which may lead to some degree of misclassification, particularly amongst the complete initial series group. A portion of these individuals could have received one shot of an initial series, failed to complete the series, but then received one “booster” shot later on. To address this, we conducted a sensitivity analysis by using a survey question about “the year of the most recent shot received”. We specifically isolated those who reported two shots, with their most recent shot in 2022, potentially indicating a booster dose. We reran the analysis including this small group as a separate category, but it did not affect the results in any meaningful way. Behavioral data suggests that individuals who are moderate vaccine supporters or opponents were more likely to experience a decrease in vaccine confidence as the pandemic progressed. COVID-19 vaccination rates decrease as support for vaccines decreases. This indicates that the number of people who did not complete their primary vaccine series and then received a booster dose may be relatively small [39].

Finally, surveys did not include the number of past infections per respondent, information on anti-viral medication, or when the worst symptomatic infection occurred in relation to the most prevalent virus variant. If available, data on participants’ use of antiviral treatments (i.e., nirmatrelvir) would likely have a greater impact on our symptom-severity analysis, as research on the effectiveness of antiviral drugs for preventing long COVID has been mixed [40,41,42]. An increase in the number of times a patient has had a COVID infection is associated with an increase in the likelihood of experiencing long-term symptoms, thus participant reinfection statistics are more likely to influence our long-COVID analysis [43]. A lack of variant information may affect our findings [11]. Finally, our study relies on self-reported data, which can be subject to recall bias or misclassification bias.

## 5. Conclusions

These findings suggest that receipt of additional booster doses is associated with a reduced likelihood of both experiencing a severe COVID-19 infection and with experiencing long COVID. The continuation of booster vaccination campaigns may be necessary to prevent long COVID, but additional time-ordered studies should be conducted to further evaluate the impact of boosters on long COVID.

## Figures and Tables

**Table 1:** Descriptive Statistics of Population that tested Positive for COVID-19.

Characteristics	Total		None/Incomplete Dose		Complete Initial Series		≥1 Booster Dose		Chi-Square Test *p*-Value
	Unweighted n (%)	Weighted n (%)	Unweighted n (%)	Weighted n (%)	Unweighted n (%)	Weighted n (%)	Unweighted n (%)	Weighted n (%)	
**Total**	9521	95,236,019 (100%)	2092 (22.0%)	22,644,032 (23.9%)	2981 (31.3%)	3,174,379 (33.6%)	4448 (46.7%)	40,202,008 (42.5%)	
**Age**									
18–29 years old	1588 (16.7%)	22,624,581 (24.0%)	498 (23.8%)	7,256,483 (32.1%)	563 (18.9%)	8,350,606 (26.4%)	527 (11.9%)	7,017,492 (17.5%)	*p* < 0.001
30–39 years old	1945 (20.5%)	19,478,712 (20.6%)	502 (24.0%)	5,441,657 (24.0%)	733 (24.7%)	7,405,471 (23.4%)	710 (16.0%)	6,631,584 (16.5%)	
40–49 years old	1623 (17.1%)	16,677,646 (17.7%)	351 (16.8%)	3,799,570 (16.8%)	571 (19.2%)	6,061,386 (19.2%)	701 (15.8%)	6,816,690 (17.0%)	
50–59 years old	1496 (15.7%)	15,013,554 (15.9%)	308 (14.7%)	3,029,090 (13.4%)	473 (15.9%)	5,061,604 (16.0%)	715 (16.1%)	6,922,859 (17.3%)	
60–69 years old	1531 (16.1%)	12,068,566 (12.8%)	256 (12.2%)	2,002,165 (8.8%)	370 (12.4%)	2,997,236 (9.50%)	905 (20.4%)	7,069,165 (17.6%)	
70–79 years old	937 (9.9%)	6,235,587 (6.6%)	118 (5.6%)	737,051 (3.3%)	196 (6.6%)	1,359,402 (4.3%)	623 (14.0%)	4,139,134 (10.3%)	
80–85 years old	386 (4.1%)	2,349,503 (2.5%)	58 (2.8%)	375,889 (1.7%)	68 (2.3%)	437,318 (1.4%)	260 (5.9%)	1,536,296 (3.8%)	
**Sex**									
Male	4217 (44.3%)	44,943,940 (47.5%)	987 (47.2%)	11,538,925 (51.0%)	1349 (45.3%)	1,5385,050 (48.5%)	1881 (42.3%)	18,019,965 (44.9%)	*p* < 0.001
Female	5302 (55.7%)	49,618,876 (52.5%)	1105 (52.8%)	11,105,106 (49.0%)	1632 (54.8%)	16,357,329 (51.5%)	2565 (57.7%)	22,156,440 (55.1%)	
**Race and Ethnicity**									
Non-Hispanic White	7107 (74.7%)	69,185,195 (73.1%)	1626 (77.7%)	17,361,436 (76.7%)	2117 (71.0%)	22,270,505 (70.2%)	3364 (75.6%)	29,553,254 (73.5%)	*p* < 0.001
Non-Hispanic Black	1004 (10.6%)	10,257,068 (10.8%)	244 (11.7%)	2,696,609 (11.9%)	377 (12.7%)	4,006,676 (12.6%)	383 (8.6%)	3,553,783 (8.80%)	
Non-Hispanic Asian	514 (5.4%)	4,994,154 (5.3%)	23 (1.1%)	250,890 (1.1%)	145 (4.9%)	1,570,215 (4.9%)	346 (7.8%)	3,173,049 (7.9%)	
Hispanic	571 (6.0%	6,694,542 (7.1%)	112 (5.4%)	1,406,430 (6.2%)	231 (7.8%)	2,639,292 (8.3%)	228 (5.1%)	2,648,821 (6.6%)	
Other or multiple	325 (3.4%)	3,457,460 (3.7%)	87 (4.2%)	928,668 (4.1%)	111 (3.7%)	1,255,691 (4.0%	127 (2.9%)	1,273,101 (3.5%)	
**PIR**									
<1.25	1193 (12.5%)	11,313,800 (12.0%)	408 (19.5%)	4,032,941 (17.8%)	385 (12.9%)	3,690,288 (11.6%)	400 (9.0%)	3,590,570 (8.90%)	*p* < 0.001
1.25 < 3.0	2618 (27.5%)	27,023,572 (28.6%)	785 (37.5%)	8,751,032 (38.6%)	872 (29.3%)	9,491,440 (29.9%)	961 (21.6%)	8,781,100 (21.8%)	
≥3.0	5710 (60.0%)	56,251,047 (59.5%)	899 (43.0%)	9,860,059 (43.5%)	1724 (57.8%)	18,560,651 (58.5%)	3087 (69.4%)	27,830,338 (69.2%)	
**Education Level**									
HS Degree or less	2887 (30.4%)	32,266,305 (34.3%)	943 (45.3%)	11,068,397 (49.2%)	958 (32.3%)	11,210,762 (35.5%)	986 (22.%)	9,987,145 (25.0%)	*p* < 0.001
Some college	1456 (15.4%)	15,998,989 (17.0%)	367 (17.6%)	4,114,654 (18.3%)	519 (17.5%)	5,924,244 (18.8%)	570 (12.9%)	5,960,091 (14.9%)	
Associate’s degree	1309 (13.8%)	13,073,488 (13.9%)	334 (16.1%)	3,573,865 (15.9%)	412 (13.9%)	4,444,417 (14.1%)	563 (12.7%)	5,055,206 (12.6%)	
Bachelor’s degree	2391 (25.2%)	20,798,706 (22.1%)	325 (15.6%)	2,853,734 (12.7%)	756 (25.5%)	7,069,216 (22.4%)	1310 (29.5%)	10,875,757 (27.2%)	
Graduate degree	1441 (15.2%)	11,949,916 (12.7%)	111 (5.3%)	885,796 (3.9%)	325 (10.9%)	2,921,607 (9.3%)	1005 (22.7%)	8,142,513 (20.3%)	
**Smoking History**	3177 (33.8%)	29,282,076 (31.3%)	812 (39.1%)	8,137,128 (36.2%)	932 (31.7%)	9,166,416 (29.3%)	1433 (32.6%)	11,978,532 (30.2%)	*p* < 0.001
**BMI Category**									
Underweight	116 (1.2%)	1,259,139 (1.4%)	36 (1.8%)	473,446 (2.1%)	31 (1.1%)	363,881 (1.2%)	49 (1.1%)	421,812 (1.1%)	*p* < 0.001
Healthy weight	2772 (29.7%)	27,811,823 (29.9%)	537 (26.1%)	5,889,124 (26.5%)	832 (28.4%)	9,201,797 (29.4%)	1403 (32.2%)	12,720,902 (32.2%)	
Overweight	3174 (34.0%)	30,854,701 (33.2%)	684 (33.3%)	7,373,511 (33.1%)	1004 (34.2%)	10,334,828 (33.1%)	1486 (34.1%)	13,146,362 (33.3%)	
Obese	3288 (35.2%)	33,068,665 (35.6%)	797 (38.8%)	8,516,967 (38.3%)	1067 (36.4%)	11,356,079 (36.3%)	1424 (32.7%)	13,195,619 (33.4%)	
**Prior Medical Conditions:**									
Anxiety	2897 (30.6%)	30,023,346 (31.9%)	627 (30.1%)	7,037,013 (31.1%)	905 (30.5%)	9,944,503 (31.4%)	1365 (30.9%)	13,041,830 (32.6%)	*p* = 0.510
Depression	1022 (10.8%)	10,291,087 (11.0%)	251 (12.1%)	2,709,017 (12.0%)	336 (11.3%)	3,449,985 (10.9%)	435 (9.9%)	4,132,085 (10.4%)	*p* = 0.216
COPD	434 (5.6%)	3,593,163 (3.8%)	103 (4.9%)	895,666 (4.0%)	118 (4.0%)	1,073,333 (3.4%)	213 (4.8%)	1,624,164 (4.0%)	*p* = 0.376
Asthma	922 (9.7%)	9,231,866 (9.8%)	215 (10.3%)	2,415,276 (10.7%)	273 (31.3%)	2,933,804 (9.3%)	434 (9.8%)	3,882,786 (9.7%)	*p* = 0.362
Diabetes	887 (9.3%)	8,102,918 (8.6%)	176 (8.4%)	1,679,913 (7.4%)	218 (7.3%)	2,067,628 (6.5%)	493 (11.1%)	4,355,378 (10.8%)	*p* < 0.001
Immunocompromised	510 (5.4%)	4,642,583 (4.9%)	114 (5.5%)	1,012,099 (4.5%)	126 (4.2%)	1,211,847 (3.8%)	270 (6.1%)	2,418,638 (6.0%)	*p* < 0.001
**Symptom Severity**									
Asymptomatic or Mild	4714 (49.5%)	46,185,210 (48.8%)	954 (45.6%)	10,400,234 (45.9%)	1406 (47.2%)	14,992,772 (47.2%)	2354 (52.9%)	20,792,203 (51.7%)	*p* < 0.001
Moderate or Severe	4807 (50.5%)	48,403,210 (51.2%)	1138 (54.4%)	12,243,798 (54.1%)	1575 (52.8%)	16,749,607 (52.8%)	2094 (47.1%)	19,409,805 (48.3%)	
**Long COVID**	1672 (17.6%)	16,474,510 (17.4%)	448 (21.4%)	4,550,677 (20.1%)	539 (18.1%)	5,677,468 (17.9%)	685 (15.4%)	6,246,365 (15.5%)	*p* < 0.001
**Brand of 1st vaccination**									
Pfizer	4060 (53.9%)	39,950,068 (54.6%)	94 (58.0%)	1,098,834 (59.6%)	1609 (54.2%)	17,585,696 (55.4%)	2357 (53.6%)	21,265,538 (52.9%)	
Moderna	2861 (38.0%)	26,937,385 (36.5%)	68 (42.0%)	716,735 (38.9%)	1056 (35.6%)	10,885,777 (34.3%)	1737 (39.5%)	15,334,874 (38.1%)	
Unsure, but 2 doses required	23 (0.3%)	249,800 (0.3%)	3 (0.0%)	26,770 (1.5%)	11 (0.2%)	139,787 (0.4%)	9 (0.1%)	83,243 (0.2%)	
Johnson	608 (8.1%)	6,237,348 (8.5%)	—	*—*	305 (10.3%)	3,131,119 (9.8%)	303 (6.9%)	3,106,229 (7.7%)	
Novavax	2 (0.0%)	13,325 (0.0%)	—	*—*	—	—	2 (0.0%)	13,325 (0.0%)	
Other Brand/Don’t Know	40 (0.5%)	398,800 (0.5%)	—	*—*	—	—	40 (0.9%)	398,800 (1.0%)	

**Table 2:** Weighted Logistic Regression Examining Associations with Moderate/Severe Symptom Severity of Initial Infection.

Predictors	Unadjusted		Adjusted	
	OR (95% CI)	*p* Value	aOR (95% CI)	*p* Value
**COVID-19 vaccination status**				
Received no or incomplete dosage of vaccine	*Ref*		*Ref*	
Received initial dose of vaccine	0.95 (0.83–1.09)	0.46	0.95 (0.82–1.10)	0.46
Received 1 or more booster shots	0.79 (0.70–0.90)	<0.001	0.78 (0.68–0.90)	<0.001
**Age**				
18–29 years old	*Ref*		*Ref*	
30–39 years old	1.02 (0.87–1.20)	0.79	1.0 (0.85–1.18)	0.99
40–49 years old	1.13 (0.96–1.34)	0.16	1.1 (0.92–1.32)	0.28
50–59 years old	1.02 (0.86–1.20)	0.83	1.0 (0.83–1.19)	0.96
60–69 years old	0.89 (0.75–1.05)	0.16	0.89 (0.74–1.06)	0.19
70–79 years old	0.68 (0.56–0.82)	<0.001	0.7 (0.56–0.86)	0.001
80–85 years old	0.76 (0.59–0.99)	0.04	0.81 (0.61–1.08)	0.15
**Sex**				
Male	*Ref*		*Ref*	
Female	1.33 (1.21–1.46)	<0.001	1.27 (1.14–1.40)	<0.001
**Race and Ethnicity**				
Non-Hispanic White	*Ref*		*Ref*	
Non-Hispanic Black	0.78 (0.66–0.92)	0.003	0.8 (0.67–0.96)	0.01
Non-Hispanic Asian	0.7 (0.57–0.86)	0.001	0.82 (0.66–1.01)	0.06
Hispanic	1.12 (0.92–1.37)	0.26	1.27 (1.02–1.59)	0.03
Other/Multiple	1.1 (0.82–1.50)	0.52	0.98 (0.73–1.33)	0.89
**Poverty Income Ratio**				
<1.25	*Ref*		*Ref*	
1.25 < 3.0	1.15 (0.97–1.37)	0.11	1.27 (1.05–1.52)	0.01
≥3.0	1.08 (0.93–1.26)	0.33	1.29 (1.08–1.53)	0.01
**Education Level**				
HS degree or less	*Ref*		*Ref*	
Some college	1.12 (0.97–1.30)	0.13	1.1 (0.95–1.29)	0.21
Associate’s degree	1.23 (1.05–1.44)	0.01	1.22 (1.03–1.44)	0.02
Bachelor’s degree	1.03 (0.90–1.18)	0.64	1.09 (0.94–1.27)	0.26
Graduate degree	1.04 (0.89–1.20)	0.64	1.12 (0.94–1.34)	0.21
**Smoking History**	1.08 (0.98–1.19)	0.14	1.04 (0.93–1.16)	0.49
**BMI Category**				
Underweight	1.03 (0.66–1.59)	0.91	0.97 (0.61–1.53)	0.88
Healthy weight	*Ref*		*Ref*	
Overweight	0.97 (0.86–1.09)	0.61	1.04 (0.92–1.18)	0.55
Obese	1.25 (1.11–1.41)	<0.001	1.2 (1.05–1.36)	0.01
**Prior Medical Conditions:**				
Anxiety	1.55 (1.39–1.73)	<0.001	1.32 (1.17–1.50)	<0.001
Depression	1.7 (1.43–2.01)	<0.001	1.3 (1.07–1.57)	0.01
COPD	1.4 (1.11–1.77)	0.01	1.23 (0.94–1.61)	0.13
Asthma (current)	1.59 (1.35–1.88)	<0.001	1.39 (1.17–1.66)	<0.001
Diabetes	1.2 (1.02–1.42)	0.03	1.26 (1.05–1.52)	0.01
Immunocompromised	1.65 (1.32–2.06)	<0.001	1.35 (1.07–1.71)	0.01

**Table 3:** Weighted Logistic Regression Examining Associations with Long COVID.

Predictors	Unadjusted		Adjusted	
	OR (95% CI)	*p* Value	aOR (95% CI)	*p* Value
**COVID-19 vaccination status**				
Received no or incomplete dosage of vaccine	*Ref*		*Ref*	
Received initial dose of vaccine	0.87 (0.73–1.02)	0.09	0.89 (0.74–1.07)	0.22
Received 1 or more booster shots	0.73 (0.62–0.86)	<0.001	0.76 (0.63–0.91)	0.003
**Symptom Severity**				
Mild or no symptoms	*Ref*		*Ref*	
Moderate or severe symptoms	4.41 (3.80–5.11)	<0.001	3.88 (3.32–4.54)	<0.001
**Age**				
18–29 years old	*Ref*		*Ref*	
30–39 years old	1.64 (1.34–2.02)	<0.001	1.8 (1.44–2.24)	<0.001
40–49 years old	1.68 (1.36–2.07)	<0.001	1.77 (1.40–2.23)	<0.001
50–59 years old	1.61 (1.29–1.99)	<0.001	1.83 (1.43–2.34)	<0.001
60–69 years old	1.67 (1.36–2.05)	<0.001	2.06 (1.62–2.61)	<0.001
70–79 years old	1.14 (0.88–1.48)	0.309	1.53 (1.11–2.09)	0.009
80–85 years old	1.16 (0.80–1.69)	0.445	1.69 (1.11–2.56)	0.01
**Sex**				
Male	*Ref*		*Ref*	
Female	1.68 (1.47–1.91)	<0.001	1.44 (1.24–1.66)	<0.001
**Race and Ethnicity**				
Non-Hispanic White	*Ref*		*Ref*	
Non-Hispanic Black	0.83 (0.68–1.02)	0.08	0.78 (0.63–0.98)	0.03
Non-Hispanic Asian	0.38 (0.27–0.54)	<0.001	0.55 (0.38–0.80)	0.002
Hispanic	1.17 (0.90–1.53)	0.25	1.21 (0.89–1.65)	0.23
Other/Multiple	1.38 (1.02–1.87)	0.04	1.28 (0.88–1.84)	0.19
**Poverty Income Ratio**				
<1.25	*Ref*		*Ref*	
1.25 < 3.0	1 (0.82–1.23)	0.97	1.08 (0.87–1.34)	0.47
≥3.0	0.74 (0.61–0.89)	0.002	0.91 (0.73–1.13)	0.38
**Education Level**				
HS degree or less	*Ref*		*Ref*	
Some college	1.11 (0.91–1.36)	0.29	1.08 (0.86–1.34)	0.52
Associate’s degree	1.38 (1.13–1.67)	0.001	1.4 (1.13–1.72)	0.002
Bachelor’s degree	0.88 (0.74–1.05)	0.15	1.06 (0.86–1.30)	0.6
Graduate degree	0.75 (0.61–0.92)	0.006	0.87 (0.68–1.12)	0.27
**Smoking History**	1.25 (1.10–1.42)	0.001	1.03 (0.89–1.18)	0.71
**BMI Category**				
Underweight	0.79 (0.42–1.46)	0.45	0.74 (0.39–1.41)	0.36
Healthy weight	*Ref*		*Ref*	
Overweight	1.14 (0.96–1.34)	0.13	1.13 (0.94–1.36)	0.19
Obese	1.88 (1.60–2.20)	<0.001	1.49 (1.23–1.78)	<0.001
**Prior Medical Conditions:**				
Anxiety	1.87 (1.65–2.11)	<0.001	1.49 (1.26–1.77)	<0.001
Depression	2.12 (1.76–2.54)	<0.001	1.23 (0.97–1.55)	0.09
COPD	2.43 (1.88–3.15)	<0.001	1.42 (1.07–1.89)	0.02
Asthma (current)	2.28 (1.88–2.77)	<0.001	1.65 (1.33–2.05)	<0.001
Diabetes	1.31 (1.05–1.64)	0.02	0.95 (0.73–1.22)	0.68
Immunocompromised	2.3 (1.83–2.90)	<0.001	1.43 (1.12–1.82)	0.004

## Data Availability

The National Health Interview Survey (NHIS) data that support the findings of this study are publicly available at https://www.cdc.gov/nchs/nhis/data-questionnaires-documentation.htm (accessed on 15 August 2023).

## References

[1] Feikin D.R., Abu-Raddad L.J., Andrews N., Davies M.-A., Higdon M.M., Orenstein W.A., Patel M.K. (2022). Assessing Vaccine Effectiveness against Severe COVID-19 Disease Caused by Omicron Variant. Report from a Meeting of the World Health Organization. Vaccine.

[2] Huang Y.-Z., Kuan C.-C. (2022). Vaccination to Reduce Severe COVID-19 and Mortality in COVID-19 Patients: A Systematic Review and Meta-Analysis. Eur. Rev. Med. Pharmacol. Sci..

[3] Adil M.T., Rahman R., Whitelaw D., Jain V., Al-Taan O., Rashid F., Munasinghe A., Jambulingam P. (2021). SARS-CoV-2 and the Pandemic of COVID-19. Postgrad. Med. J..

[4] Salzberger B., Buder F., Lampl B., Ehrenstein B., Hitzenbichler F., Holzmann T., Schmidt B., Hanses F. (2021). Epidemiology of SARS-CoV-2. Infection.

[5] WHO Number of COVID-19 Cases Reported to WHO. https://data.who.int/dashboards/covid19/cases?n=c.

[6] WHO United States of America Situation. https://covid19.who.int/region/amro/country/us.

[7] United Health Foundation (2022). Annual Report 2022.

[8] Ahmad F.B., Cisewski J.A., Xu J., Anderson R.N. (2023). COVID-19 Mortality Update—United States, 2022. MMWR Morb. Mortal. Wkly. Rep..

[9] Tsampasian V., Elghazaly H., Chattopadhyay R., Debski M., Naing T.K.P., Garg P., Clark A., Ntatsaki E., Vassiliou V.S. (2023). Risk Factors Associated with Post-COVID-19 Condition: A Systematic Review and Meta-Analysis. JAMA Intern. Med..

[10] Gao P., Liu J., Liu M. (2022). Effect of COVID-19 Vaccines on Reducing the Risk of Long COVID in the Real World: A Systematic Review and Meta-Analysis. Int. J. Environ. Res. Public. Health.

[11] Perlis R.H., Santillana M., Ognyanova K., Safarpour A., Lunz Trujillo K., Simonson M.D., Green J., Quintana A., Druckman J., Baum M.A. (2022). Prevalence and Correlates of Long COVID Symptoms Among US Adults. JAMA Netw. Open.

[12] Brannock M.D., Chew R.F., Preiss A.J., Hadley E.C., Redfield S., McMurry J.A., Leese P.J., Girvin A.T., Crosskey M., Zhou A.G. (2023). Long COVID Risk and Pre-COVID Vaccination in an EHR-Based Cohort Study from the RECOVER Program. Nat. Commun..

[13] Ford N.D., Slaughter D., Edwards D., Dalton A., Perrine C., Vahratian A., Saydah S. (2023). Long COVID and Significant Activity Limitation Among Adults, by Age—United States, June 1–13, 2022, to June 7–19, 2023. MMWR Morb. Mortal. Wkly. Rep..

[14] Watanabe A., Iwagami M., Yasuhara J., Takagi H., Kuno T. (2023). Protective Effect of COVID-19 Vaccination against Long COVID Syndrome: A Systematic Review and Meta-Analysis. Vaccine.

[15] Notarte K.I., Catahay J.A., Velasco J.V., Pastrana A., Ver A.T., Pangilinan F.C., Peligro P.J., Casimiro M., Guerrero J.J., Gellaco M.M.L. (2022). Impact of COVID-19 Vaccination on the Risk of Developing Long-COVID and on Existing Long-COVID Symptoms: A Systematic Review. eClinicalMedicine.

[16] Menni C., Valdes A.M., Polidori L., Antonelli M., Penamakuri S., Nogal A., Louca P., May A., Figueiredo J.C., Hu C. (2022). Symptom Prevalence, Duration, and Risk of Hospital Admission in Individuals Infected with SARS-CoV-2 during Periods of Omicron and Delta Variant Dominance: A Prospective Observational Study from the ZOE COVID Study. Lancet.

[17] Chemaitelly H., Ayoub H.H., Tang P., Coyle P., Yassine H.M., Al Thani A.A., Al-Khatib H.A., Hasan M.R., Al-Kanaani Z., Al-Kuwari E. (2023). Long-Term COVID-19 Booster Effectiveness by Infection History and Clinical Vulnerability and Immune Imprinting: A Retrospective Population-Based Cohort Study. Lancet Infect. Dis..

[18] (2022). National Center for Health Statistics National Health Interview Survey. https://www.cdc.gov/nchs/nhis/2022nhis.htm.

[19] Division of Health Interview Statistics National Center for Health Statistics Hyattsville, Maryland National Health Interview Survey 2022 Survey Description. ftp.cdc.gov/pub/Health_Statistics/NCHS/Dataset_Documentation/NHIS/2022/srvydesc-508.pdf.

[20] Romeiser J.L., Morley C.P., Singh S.M. (2023). COVID-19 Symptom Load as a Risk Factor for Chronic Pain: A National Cross-Sectional Study. PLoS ONE.

[21] Byambasuren O., Stehlik P., Clark J., Alcorn K., Glasziou P. (2023). Effect of Covid-19 Vaccination on Long Covid: Systematic Review. BMJ Med..

[22] Piernas C., Patone M., Astbury N.M., Gao M., Sheikh A., Khunti K., Shankar-Hari M., Dixon S., Coupland C., Aveyard P. (2022). Associations of BMI with COVID-19 Vaccine Uptake, Vaccine Effectiveness, and Risk of Severe COVID-19 Outcomes after Vaccination in England: A Population-Based Cohort Study. Lancet Diabetes Endocrinol..

[23] Cavanaugh A.M., Spicer K.B., Thoroughman D., Glick C., Winter K. (2021). Reduced Risk of Reinfection with SARS-CoV-2 After COVID-19 Vaccination—Kentucky, May–June 2021. MMWR Morb. Mortal. Wkly. Rep..

[24] Meeraus W., Stuurman A.L., Durukal I., Conde-Sousa E., Lee A., Maria A.S., Furtado B.E., Ouwens M., Gray C.M., Valverde D.A. (2023). COVID-19 Vaccine Booster Doses Provide Increased Protection against COVID-19 Hospitalization Compared with Previously Vaccinated Individuals: Interim Findings from the REFORCO-Brazil Real-World Effectiveness Study during Delta and Omicron. Vaccine.

[25] Marra A.R., Kobayashi T., Callado G.Y., Pardo I., Gutfreund M.C., Hsieh M.K., Lin V., Alsuhaibani M., Hasegawa S., Tholany J. (2023). The Effectiveness of COVID-19 Vaccine in the Prevention of Post-COVID Conditions: A Systematic Literature Review and Meta-Analysis of the Latest Research. Antimicrob. Steward. Healthc. Epidemiol..

[26] Marra A.R., Sampaio V.S., Ozahata M.C., Lopes R., Brito A.F., Bragatte M., Kalil J., Miraglia J.L., Malheiro D.T., Guozhang Y. (2023). Risk Factors for Long Coronavirus Disease 2019 (Long COVID) among Healthcare Personnel, Brazil, 2020–2022. Infect. Control Hosp. Epidemiol..

[27] Azzolini E., Levi R., Sarti R., Pozzi C., Mollura M., Mantovani A., Rescigno M. (2022). Association Between BNT162b2 Vaccination and Long COVID After Infections Not Requiring Hospitalization in Health Care Workers. JAMA.

[28] Chaichana U., Man K.K.C., Chen A., Wong I.C.K., George J., Wilson P., Wei L. (2023). Definition of Post–COVID-19 Condition Among Published Research Studies. JAMA Netw. Open.

[29] Vaughan L., Veruttipong D., Shaw J.G., Levy N., Edwards L., Winget M. (2021). Relationship of Socio-Demographics, Comorbidities, Symptoms and Healthcare Access with Early COVID-19 Presentation and Disease Severity. BMC Infect. Dis..

[30] Durstenfeld M.S., Peluso M.J., Peyser N.D., Lin F., Knight S.J., Djibo A., Khatib R., Kitzman H., O’Brien E., Williams N. (2023). Factors Associated with Long COVID Symptoms in an Online Cohort Study. Open Forum Infect. Dis..

[31] Jacobs E.T., Cordova-Marks F.M., Farland L.V., Ernst K.C., Andrews J.G., Vu S., Heslin K.M., Catalfamo C., Chen Z., Pogreba-Brown K. (2023). Understanding Low COVID-19 Booster Uptake among US Adults. Vaccine.

[32] Neely S.R., Hao F. (2023). Breakthrough COVID-19 Infections and Perceived Vaccine Effectiveness. Vaccine.

[33] Stein C., Nassereldine H., Sorensen R.J.D., Amlag J.O., Bisignano C., Byrne S., Castro E., Coberly K., Collins J.K., Dalos J. (2023). Past SARS-CoV-2 Infection Protection against Re-Infection: A Systematic Review and Meta-Analysis. Lancet.

[34] Nordström P., Ballin M., Nordström A. (2022). Risk of SARS-CoV-2 Reinfection and COVID-19 Hospitalisation in Individuals with Natural and Hybrid Immunity: A Retrospective, Total Population Cohort Study in Sweden. Lancet Infect. Dis..

[35] Wisnivesky J.P., Govindarajulu U., Bagiella E., Goswami R., Kale M., Campbell K.N., Meliambro K., Chen Z., Aberg J.A., Lin J.J. (2022). Association of Vaccination with the Persistence of Post-COVID Symptoms. J. Gen. Intern. Med..

[36] Nayyerabadi M., Fourcade L., Joshi S.A., Chandrasekaran P., Chakravarti A., Massé C., Paul M.-L., Houle J., Boubekeur A.M., DuSablon C. (2023). Vaccination after Developing Long COVID: Impact on Clinical Presentation, Viral Persistence, and Immune Responses. Int. J. Infect. Dis..

[37] Limbu Y.B., Gautam R.K. (2023). The Determinants of COVID-19 Vaccination Intention: A Meta-Review. Front. Public Health.

[38] Sprengholz P., Betsch C. (2021). Previous SARS-CoV-2 Infection Is Linked to Lower Vaccination Intentions. J. Med. Virol..

[39] Sobierajski T., Rzymski P., Wanke-Rytt M. (2023). Impact of the COVID-19 Pandemic on Attitudes toward Vaccination: Representative Study of Polish Society. Vaccines.

[40] Durstenfeld M.S., Peluso M.J., Lin F., Peyser N.D., Isasi C., Carton T.W., Henrich T.J., Deeks S.G., Olgin J.E., Pletcher M.J. (2024). Association of Nirmatrelvir for Acute SARS-CoV-2 Infection with Subsequent Long COVID Symptoms in an Observational Cohort Study. J. Med. Virol..

[41] Xie Y., Choi T., Al-Aly Z. (2023). Association of Treatment with Nirmatrelvir and the Risk of Post–COVID-19 Condition. JAMA Intern. Med..

[42] Geng L.N., Bonilla H., Hedlin H., Jacobson K.B., Tian L., Jagannathan P., Yang P.C., Subramanian A.K., Liang J.W., Shen S. (2024). Nirmatrelvir-Ritonavir and Symptoms in Adults with Postacute Sequelae of SARS-CoV-2 Infection: The STOP-PASC Randomized Clinical Trial. JAMA Intern. Med..

[43] Kuang S., Earl S., Clarke J., Zakaria D., Demers A., Aziz S. (2023). Experiences of Canadians with Long-Term Symptoms Following COVID-19.

